# Association of biological age acceleration with all-cause and cardiovascular mortality in HSV-positive adults: A population-based longitudinal cohort study

**DOI:** 10.1371/journal.pone.0334621

**Published:** 2025-10-14

**Authors:** Jun Wei, Yuefeng Li, Yang Liu

**Affiliations:** 1 School of Basic Medical Sciences, Jilin Medical University, Jilin, China; 2 Edinburgh Medical School: Biomedical Sciences, College of Medicine and Veterinary Medicine, The University of Edinburgh, Edinburgh, United Kingdom; 3 Zhejiang University-University of Edinburgh Institute, Zhejiang University School of Medicine, Haining, China; Rush University, UNITED STATES OF AMERICA

## Abstract

**Background:**

Biological age acceleration reflects physiological aging and its link to mortality in HSV-infected adults is unclear.

**Methods:**

We analyzed data from 16,065 HSV-seropositive adults aged 20–59 years from the NHANES 1999–2016 cycles (mean age: 35.4 ± 8.5 years). The data were collected in the United States. Biological age acceleration and Phenotypic age acceleration were calculated as residuals from regressing KDM-based biological age and PhenoAge on chronological age, respectively. The mean (SD) values were –10.9 (10.4) and –3.4 (4.6) years. Over a median follow-up of 139 months, 551 all-cause and 131 cardiovascular deaths occurred. Weighted Cox proportional hazards models were used to evaluate associations between biological age acceleration and mortality. Nonlinear associations and potential threshold effects were assessed using smooth curve fitting based on generalized additive models. Subgroup and sensitivity analyses confirmed the robustness of the results.

**Results:**

Both biological age acceleration and Phenotypic age acceleration were significantly associated with increased all-cause and cardiovascular mortality. Among individuals with Phenotypic age acceleration > –1.8, each 5-year increase was associated with a 68% higher risk of all-cause mortality (HR: 1.68; 95% CI: 1.47–1.92; *P* < 0.001). For biological age acceleration > 3.14, each 5-year increase was associated with a 16% higher risk (HR: 1.16; 95% CI: 1.03–1.30; *P* = 0.0133). Results remained consistent across subgroups and in sensitivity analyses.

**Conclusion:**

In a cohort of HSV-seropositive adults in the United States, biological age acceleration, particularly Phenotypic Age acceleration, was significantly associated with increased risks of all-cause and cardiovascular mortality.

## 1. Introduction

Herpes simplex viruses (HSV), including HSV type 1 (HSV-1) and type 2 (HSV-2), are members of the Herpesviridae family and share a linear double-stranded DNA genome enclosed in an icosahedral capsid [1]. HSV-1 is primarily transmitted through oral contact, whereas HSV-2 is predominantly spread through sexual transmission, although both viruses are capable of establishing infections at either site [2]. Globally, HSV infections are highly prevalent. As of 2016, an estimated 491.5 million individuals were living with HSV-2, accounting for roughly 13% of the world’s population [3], while HSV-1 is even more widespread, often acquired in early childhood. Following initial exposure, HSV establishes lifelong latency in the host and can periodically reactivate, leading to clinical manifestations such as oral or genital ulcers, herpetic keratitis, and in some cases, severe complications like encephalitis [4,5].

With global populations aging rapidly, increasing attention has been directed toward understanding the biological mechanisms underlying aging and its impact on health outcomes. Chronological age (CA), although widely used, does not fully capture interindividual differences in biological resilience, physiological decline, or disease susceptibility [6]. In contrast, biological age (BA) offers a more refined estimate of functional deterioration, reflecting heterogeneity in health status beyond simply the number of years lived [7,8]. Prior research has linked accelerated biological aging to a wide range of adverse outcomes, including frailty, chronic disease, and increased mortality risk [9]. To quantify BA, several algorithms have been developed based on clinical biomarkers or composite indices. Commonly used methods include homeostatic dysregulation (HD) [10], the Klemera–Doubal method (KDM) [11], phenotypic age (PA) [12], and allostatic load (AL) [13], all of which have shown robust associations with adverse health outcomes in population-based analyses.

Therefore, we aimed to investigate whether biological age acceleration (defined as the residual difference between estimated BA and CA, calculated from KDM-BA and PA [14–16]) is associated with all-cause and cardiovascular mortality among HSV-positive adults. We analyzed data from the U.S. National Health and Nutrition Examination Survey (NHANES) 1999–2016. To our knowledge, this is among the first epidemiological studies to evaluate the relationship between biological aging metrics and mortality in the context of chronic HSV infection. We hypothesized that higher age acceleration would be associated with increased mortality risk, and that risk may increase more steeply beyond specific thresholds of age acceleration.

## 2. Methods

### 2.1. Study design and population

We performed a cohort analysis of HSV-seropositive participants from NHANES with mortality follow-up. NHANES is a continuous cross-sectional survey of the U.S. civilian non-institutionalized population, conducted by the National Center for Health Statistics. The survey employs a multistage probability sampling design to ensure that participants are geographically and demographically representative of the entire United States population. Participants undergo interviews, physical examinations, and laboratory tests, and the survey data can be linked to national mortality records for longitudinal follow-up. The NHANES protocol was approved by the NCHS Research Ethics Review Board, and all participants provided written informed consent.

**Fig 1:**
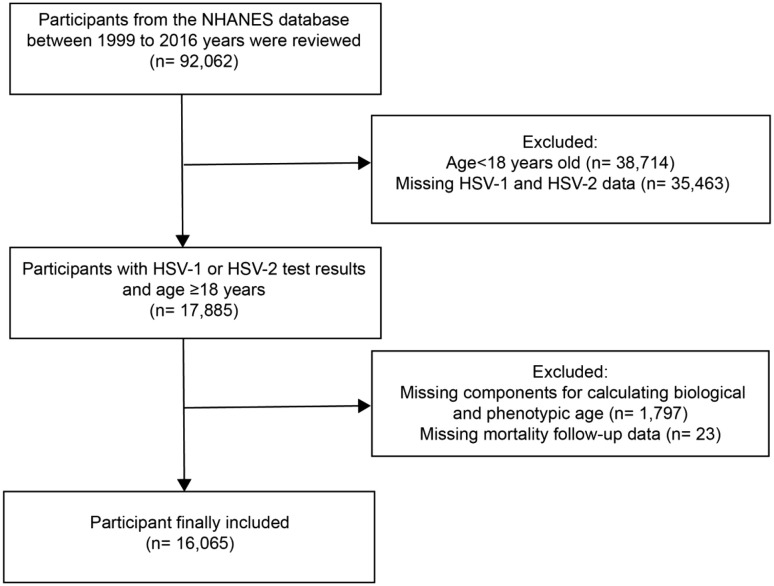
Flowchart of Sample Selection from NHANES 1999–2016. The flowchart illustrates the selection process of HSV-seropositive adults from the NHANES 1999–2016 cycles, including the inclusion and exclusion criteria, resulting in a final sample of 16,065 participants for the analysis.

We included adult participants aged 20–59 years who were seropositive for HSV-1 or HSV-2 in the NHANES 1999–2016 cycles. Participants with complete biomarker and mortality follow-up data were retained for analysis. The final sample consisted of 16,065 HSV-seropositive adults (Fig 1).

### 2.2. Biological aging measures (exposures)

Biological aging in this study was quantified using two complementary algorithms: the KDM-BA and PA. Both measures were constructed using standard clinical biomarkers available in NHANES, following previously validated protocols [17,18].

KDM-BA:

The KDM approach estimates the age at which an individual’s biomarker profile would be considered “normal” in a reference population. For each participant, KDM-BA was calculated using the following equation:


$$KDM-BA=\frac{\sum_{i=\text{1}}^{n}\left(x_{i}-q_{i}\right)\frac{k_{i}}{s_{i}^{\text{2}}}+\frac{CA}{s_{BA}^{\text{2}}}}{\sum_{i=\text{1}}^{n}{\left(\frac{k_{i}}{s_{i}}\right)}^{\text{2}}+\frac{\text{1}}{s_{BA}^{\text{2}}}}$$

where *x*_*i*_ is the observed value of biomarker *i; q*_*i*_*, k*_*i*_*, and s*_*i*_ represent the intercept, slope, and root mean squared error from regressing chronological age on biomarker *i* in the reference sample; *CA* is chronological age; and *s*_*BA*_ is a scaling factor corresponding to the variance in age explained by the biomarker set. In our analysis, the following eight markers were included: natural log-transformed C-reactive protein (CRP), serum creatinine, glycated hemoglobin (HbA1c), serum albumin, total cholesterol, blood urea nitrogen (BUN), serum alkaline phosphatase (ALP), and systolic blood pressure (SBP).

PA:

PhenoAge was derived from a Gompertz proportional hazards model that integrates chronological age and nine clinical biomarkers predictive of mortality. The algorithm converts predicted mortality risk into an equivalent “biological age” using the equation:

where the linear predictor is defined as:


$$Phenotypicage=\frac{\text{Ln}\left[-\text{0}.\text{0}\text{0}\text{5}\text{5}\text{3}*\text{Ln}\left(\frac{-\text{1}.\text{5}\text{1}\text{7}\text{1}\text{4}*\text{exp}(xb)}{\text{0}.\text{0}\text{0}\text{7}\text{6}\text{9}\text{2}\text{7}}\right)\right]}{\text{0}.\text{0}\text{9}\text{1}\text{6}\text{5}}$$

*xb*=−19.907 − 0.0336 × Albumin+0.0095 × Creatinine+0.1953 × Glucose−0.0120 × Lymphocyte% + 0.0268 × Mean Cell Volume+0.3306 × Red Cell Distribution Width+0.00188 × ALP + 0.0554 × White Blood Cell Count+0.0804 × Chronological Age.

Age Acceleration Metrics:

To capture deviations from expected aging, biological age acceleration was defined as the residual from regressing each biological age estimate (KDM-BA or PA) on chronological age. Specifically, positive residuals indicate accelerated biological aging relative to peers of the same chronological age, while negative values reflect decelerated aging. Both KDM-based biological age acceleration and PhenoAge acceleration were treated as continuous exposure variables in the primary analyses.

All calculations were performed using the BioAge R package (https://github.com/dayoonkwon/BioAge), which implements these algorithms using standardized NHANES reference data.

### 2.3. Outcome ascertainment: Mortality

The data on mortality were obtained from death certificate records in the National Death Index (NDI), which were supplied by the National Center for Health Statistics (NCHS), with updates made until December 31, 2019. The endpoints of the study included both all-cause mortality and mortality specifically related to cardiovascular issues. All-cause mortality was defined using the underlying cause of death recorded in the NDI, and cardiovascular mortality was defined according to ICD-10 underlying cause-of-death codes for heart disease (I00–I09, I11, I13, I20–I51), following NCHS documentation [19].

### 2.4. Measurement of herpes simplex virus

Blood samples were collected via venipuncture at a mobile examination center and processed according to standardized protocols. HSV-1 and HSV-2 serostatus was determined at Emory University using a type-specific enzyme immunoassay based on purified glycoproteins gG-1 and gG-2. These antigens, immobilized on nitrocellulose disks, enable differentiation between HSV-1 and HSV-2 despite cross-reactivity between herpesviruses. Serum antibodies binding to gG-1 or gG-2 were detected using peroxidase-conjugated goat anti-human IgG and a chromogenic substrate. A visible blue dot indicated seropositivity, with reactivity to gG-1 signifying HSV-1 infection and to gG-2 indicating HSV-2 infection. All participants provided written informed consent for sample use [20].

### 2.5. Covariates

We included a comprehensive set of baseline covariates as potential confounders, selected based on prior literature and their known associations with biological aging and mortality. Demographic variables included chronological age (years), sex (male or female), and race/ethnicity, categorized as Non-Hispanic White, Non-Hispanic Black, Mexican American, Other Hispanic, and Other Race. Socioeconomic factors comprised marital status (married/living with partner, widowed/divorced/separated, never married), education level (below high school, high school graduate, above high school), and the poverty income ratio (PIR), classified as poor, nearly poor, middle income, or high income, with missing data retained as a separate category.

Health behavior variables included smoking status (never, former, or current) and alcohol use (never, former, mild, moderate, or heavy), based on self-reported quantity and frequency. Physical activity was assessed using total weekly moderate-to-vigorous metabolic equivalent task (MET) minutes, and categorized as <600 MET-min/week, ≥ 600 MET-min/week, or missing. Clinical variables included body mass index (BMI, kg/m²), hypertension, diabetes mellitus, hyperlipidemia, and cardiovascular disease, all identified via physical examinations, laboratory measurements, medication use, or physician diagnosis.

We also considered impaired fasting glycaemia (IFG) and impaired glucose tolerance (IGT) as indicators of subclinical metabolic dysfunction. PA and BA were used solely to derive age acceleration metrics and were not included as independent covariates in multivariable models. HSV serotype was not adjusted for, as all participants were HSV-positive. Covariate selection was based on clinical relevance and existing evidence rather than statistical significance. Demographic and socioeconomic factors were included because they influence biomarker profiles used to construct KDM-BA and PA and are associated with survival. Health behaviors and BMI were included due to their established links with systemic inflammation and metabolic function. Hypertension and diabetes were considered potential intermediates between accelerated aging and outcomes; therefore, they were excluded from the primary models but examined in sensitivity analyses to assess robustness.

### 2.6. Statistical analysis

All statistical analyses were performed in accordance with NHANES analytic guidelines, incorporating sample weights, strata, and primary sampling units to account for the complex, multistage probability sampling design. Baseline characteristics between survivors and non-survivors were compared using weighted means and 95% confidence intervals (CIs) for continuous variables, and weighted proportions with 95% CIs for categorical variables. Between-group differences were assessed using survey-weighted linear regression for continuous variables and the Rao–Scott chi-square test for categorical variables.

Covariate-adjusted survival curves by tertiles of age acceleration were derived from fully adjusted survey-weighted Cox proportional hazards models using marginal standardization, thereby accounting for the complex sampling design. Associations between age acceleration and mortality were estimated with survey-weighted Cox proportional hazards models. Three progressively adjusted models were specified: Model 1 (unadjusted), Model 2 (adjusted for chronological age and sex), and Model 3 (fully adjusted for predefined demographic, socioeconomic, behavioral, and clinical covariates based on prior literature).

To examine potential non-linear associations, generalized additive models (GAMs) with penalized spline smoothing were used to fit smooth curves within the Cox regression framework. When a non-linear pattern was detected, inflection points were determined using a recursive algorithm, and two-piecewise Cox models were subsequently fitted to identify potential threshold effects. Stratified analyses were conducted across key subgroups, and interaction terms were tested to evaluate potential effect modification. All analyses accounted for the complex survey design and were conducted using R software (version 4.2.0) and EmpowerStats (version 4.2), with a two-sided P-value < 0.05 considered statistically significant.

## 3. Results

### 3.1. Demographic characteristics of the HSV population

A total of 16,065 participants were included in the analysis, with 15,514 survivors and 551 non-survivors. The mean age of the cohort was 35.38 years, with non-survivors being significantly older than survivors (39.67 vs. 35.23 years, *P* < 0.001). Males comprised a higher proportion of non-survivors (56.08%) compared to survivors (43.90%). Non-survivors also had a higher mean BMI (29.85 kg/m^2^ vs. 29.01 kg/m^2^, P = 0.007), and higher phenotypic age acceleration and BA. No significant difference was found in biological age acceleration between the two groups.

In terms of ethnicity, Non-Hispanic Whites represented 33.49% of the total population but 40.83% of non-survivors. Non-survivors were more likely to be widowed, divorced, or separated compared to survivors, and had higher rates of smoking, alcohol consumption, and lower physical activity. They were also more likely to live in poverty, have lower educational attainment, and suffer from chronic conditions such as hypertension, diabetes, hyperlipidemia, and cardiovascular disease.

Regarding HSV serostatus, non-survivors had a lower prevalence of HSV-1 infection (86.9% vs. 90.1%, *P* = 0.013), while HSV-2 infection was more prevalent among non-survivors (39.7% vs. 29.4%, *P* < 0.001). Detailed stratified characteristics by survival status are presented in Table 1.

**Table 1:** Baseline demographic characteristics of the HSV population stratified by survival status.

Variable	Total (n = 16,065)	Survivor (n = 15,514)	Non-survivors (n = 551)	P value
Age (years)	35.38 ± 8.49	35.23 ± 8.47	39.67 ± 7.85	<0.001
Sex				<0.001
Male	7119 (44.31%)	6810 (43.90%)	309 (56.08%)	
Female	8946 (55.69%)	8704 (56.10%)	242 (43.92%)	
BMI (kg/m2)	29.03 ± 7.07	29.01 ± 7.02	29.85 ± 8.18	0.007
PA	31.73 ± 9.67	31.47 ± 9.54	38.52 ± 10.44	<0.001
Phenotypic age acceleration	−3.49 ± 4.70	−3.58 ± 4.62	−1.27 ± 5.93	<0.001
BA	24.40 ± 10.78	24.28 ± 10.68	27.89 ± 12.79	<0.001
Biological age acceleration	−10.98 ± 10.42	−10.95 ± 10.35	−11.78 ± 12.20	0.066
Ethnicity				<0.001
Non-Hispanic White	5380 (33.49%)	5155 (33.23%)	225 (40.83%)	
Non-Hispanic Black	3765 (23.44%)	3588 (23.13%)	177 (32.12%)	
Mexican American	4027 (25.07%)	3914 (25.23%)	113 (20.51%)	
Other Hispanic	1540 (9.59%)	1523 (9.82%)	17 (3.09%)	
Other Race	1353 (8.42%)	1334 (8.60%)	19 (3.45%)	
Marital status, n (%)				<0.001
Married/Living with Partner	9943 (61.89%)	9650 (62.20%)	293 (53.18%)	
Widowed/Divorced/Separated	2097 (13.05%)	1981 (12.77%)	116 (21.05%)	
Never married	3834 (23.87%)	3705 (23.88%)	129 (23.41%)	
Missing	191 (1.19%)	178 (1.15%)	13 (2.36%)	
PIR				<0.001
Poor	3853 (23.98%)	3680 (23.72%)	173 (31.40%)	
Nearly poor	4047 (25.19%)	3899 (25.13%)	148 (26.86%)	
Middle income	3812 (23.73%)	3691 (23.79%)	121 (21.96%)	
High income	3126 (19.46%)	3067 (19.77%)	59 (10.71%)	
Missing	1227 (7.64%)	1177 (7.59%)	50 (9.07%)	
Education level, n (%)				<0.001
Below high school	1604 (9.98%)	1534 (9.89%)	70 (12.70%)	
High school	6827 (42.50%)	6538 (42.14%)	289 (52.45%)	
Above high school	7623 (47.45%)	7432 (47.91%)	191 (34.66%)	
Missing	11 (0.07%)	10 (0.06%)	1 (0.18%)	
Smoking status, n (%)				<0.001
Never	9197 (57.25%)	9014 (58.10%)	183 (33.21%)	
Former	2461 (15.32%)	2381 (15.35%)	80 (14.52%)	
Now	4395 (27.36%)	4107 (26.47%)	288 (52.27%)	
Missing	12 (0.07%)	12 (0.08%)	0 (0.00%)	
Alcohol use, n (%)				<0.001
Never	1947 (12.12%)	1902 (12.26%)	45 (8.17%)	
Former	1905 (11.86%)	1801 (11.61%)	104 (18.87%)	
Mild	3768 (23.45%)	3672 (23.67%)	96 (17.42%)	
Moderate	2497 (15.54%)	2424 (15.62%)	73 (13.25%)	
Heavy	4352 (27.09%)	4156 (26.79%)	196 (35.57%)	
Missing	1596 (9.93%)	1559 (10.05%)	37 (6.72%)	
Total physical activity (MET/week)				<0.001
<600	4326 (26.93%)	4144 (26.71%)	182 (33.03%)	
≥600	7889 (49.11%)	7682 (49.52%)	207 (37.57%)	
Missing	3850 (23.97%)	3688 (23.77%)	162 (29.40%)	
Hypertension				<0.001
No	12559 (78.21%)	12236 (78.90%)	323 (58.62%)	
Yes	3500 (21.79%)	3272 (21.10%)	228 (41.38%)	
Diabetes Mellitus				<0.001
No	13156 (86.99%)	12750 (87.38%)	406 (76.46%)	
Yes	1129 (7.47%)	1037 (7.11%)	92 (17.33%)	
IFG	458 (3.03%)	433 (2.97%)	25 (4.71%)	
IGT	380 (2.51%)	372 (2.55%)	8 (1.51%)	
Hyperlipidemia				0.010
No	5515 (34.33%)	5354 (34.51%)	161 (29.22%)	
Yes	10550 (65.67%)	10160 (65.49%)	390 (70.78%)	
Cardiovascular Diseases				<0.001
No	15616 (97.22%)	15119 (97.47%)	497 (90.20%)	
Yes	447 (2.78%)	393 (2.53%)	54 (9.80%)	
HSV-1				0.013
No	1602 (9.98%)	1530 (9.87%)	72 (13.09%)	
Yes	14451 (90.02%)	13973 (90.13%)	478 (86.91%)	
HSV-2				<0.001
No	11243 (70.24%)	10912 (70.59%)	331 (60.29%)	
Yes	4764 (29.76%)	4546 (29.41%)	218 (39.71%)	

P values were calculated using either Student’s t-test or the chi-square test. BMI, body mass index; PIR, poverty income ratio; IFG, impaired fasting glycaemia; IGT, impaired glucose tolerance; BA, biological age; PA, phenotypic age; HSV, herpes simplex viruses.

### 3.2. Nonlinear associations of biological and phenotypic age acceleration with all-cause and cardiovascular mortality

**Fig 2:**
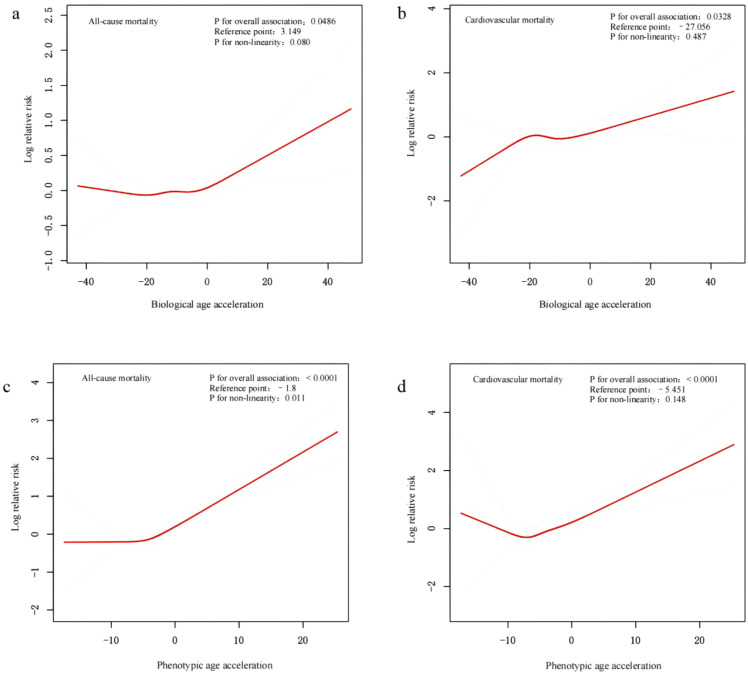
Nonlinear Associations Between Biological and Phenotypic Age Acceleration and Mortality Risks. The solid red lines represent the fitted nonlinear associations estimated using penalized spline Cox proportional hazards models, while the blue-shaded areas indicate 95% confidence intervals. All models were adjusted for the following potential confounders: chronological age (years), sex, ethnicity, marital status, poverty income ratio, education level, smoking status, alcohol use, total physical activity (MET-min/week), hypertension, and diabetes mellitus.

Restricted cubic spline and threshold effect analyses (Fig 2, Table 2) revealed distinct nonlinear associations between biological and phenotypic age acceleration and mortality outcomes. For biological age acceleration, a threshold at 3.15 was identified for all-cause mortality (Fig 2a), where the risk increased modestly beyond the inflection point (HR = 1.16, 95% CI: 1.03–1.30; *P* for non-linearity = 0.080; Table 2). In contrast, no meaningful threshold effect was observed for cardiovascular mortality (Fig 2b), and the association was linear with weak statistical significance (HR = 1.09, 95% CI: 1.01–1.18; *P* = 0.0328).

**Table 2:** Threshold effect analysis.

	Adjusted HR (95%CI)	*P* value
**All-cause mortality**		
Biological age acceleration (each 5-year increase)		
One line effect	1.04 (1.00, 1.09)	0.0486
Inflection point	3.149	
Biological age acceleration< 3.149	1.01 (0.96, 1.07)	0.6267
Biological age acceleration>3.149	1.16 (1.03, 1.30)	0.0133
P for Log-likelihood ratio	**0.080**	
Phenotypic age acceleration (each 5-year increase)		
One line effect	1.47 (1.33, 1.62)	<0.0001
Inflection point	−1.8	
Phenotypic age acceleration <−1.8	1.14 (0.93, 1.41)	0.2078
Phenotypic age acceleration > −1.8	1.68 (1.47, 1.92)	<0.0001
P for Log-likelihood ratio	**0.011**	
**Cardiovascular mortality**		
Biological age acceleration (each 5-year increase)		
One line effect	1.09 (1.01, 1.18)	0.0328
Inflection point	−27.056	
Biological age acceleration < −27.056	1.56 (0.52, 4.64)	0.4258
Biological age acceleration > −27.056	1.08 (1.00, 1.18)	0.0625
P for Log-likelihood ratio	**0.487**	
Phenotypic age acceleration (each 5-year increase)		
One line effect	1.53 (1.26, 1.85)	<0.0001
Inflection point	−5.451	
Phenotypic age acceleration <−5.451	0.82 (0.36, 1.84)	0.6283
Phenotypic age acceleration >-5.451	1.65 (1.33, 2.05)	<0.0001
P for Log-likelihood ratio	**0.148**	

Hazard ratios (HRs) with 95% confidence intervals (CIs) were estimated using Cox proportional hazards models. Both standard linear and two-piecewise linear regression models were applied to evaluate the association of Biological age acceleration and Phenotypic age acceleration with mortality outcomes. Inflection points were identified using a recursive algorithm. The P value for the log-likelihood ratio test compares the goodness-of-fit between the piecewise and standard linear models. Bold font indicates a statistically significant log-likelihood ratio test (P < 0.05).

All models were adjusted for age, sex, ethnicity, marital status, poverty income ratio, education level, smoking status, alcohol use, total physical activity (MET-min/week), hypertension, and diabetes mellitus.

Phenotypic age acceleration demonstrated stronger and more consistent associations. For all-cause mortality, an inflection point was detected at –1.8 (Fig 2c), above which risk increased significantly (HR = 1.68, 95% CI: 1.47–1.92; *P* for non-linearity = 0.011; Table 2), while no significant association was observed below this point. A similar pattern was observed for cardiovascular mortality (Fig 2d), with an inflection point at –5.45 and a higher relative risk among participants above the threshold (HR = 1.65, 95% CI: 1.33–2.05; *P* < 0.0001).

### 3.3. Tertiles of age acceleration and their link to mortality

**Fig 3:**
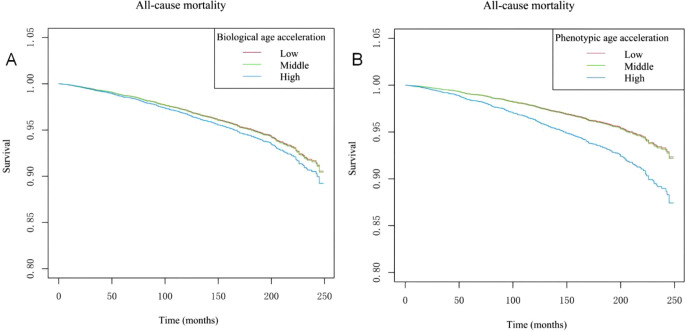
Kaplan–Meier Survival Curves for All-Cause Mortality by Tertiles of Age Acceleration. Kaplan–Meier survival curves for all-cause mortality stratified by tertiles of age acceleration after covariate adjustment. **(A)** Biological age acceleration. Individuals in the highest tertile (blue line) exhibited consistently lower survival probability compared with the middle (green line) and lowest tertiles (red line), although the separation between curves was modest. **(B)** Phenotypic age acceleration. The highest tertile showed a more pronounced decline in survival probability, with a clearer stepwise gradient across tertiles. All curves were adjusted for baseline covariates, including chronological age, sex, ethnicity, marital status, poverty income ratio, education level, smoking status, alcohol use, physical activity, hypertension, and diabetes mellitus.

Fig 3 presents the Kaplan–Meier survival curves illustrating the relationship between biological age acceleration, phenotypic age acceleration, and all-cause mortality. In Fig A, individuals with higher biological age acceleration (blue line) exhibit lower survival probabilities, with a gradual decline in survival rate compared to those in the middle (green line) and lower (red line) tertiles, although the differences between the curves are modest. In Fig B, individuals with higher phenotypic age acceleration (blue line) show a more pronounced decline in survival probability, with a clearer separation between the survival curves.

### 3.4. Stratified associations of age acceleration with mortality

Stratified analyses showed that Biological age acceleration was modestly associated with all-cause mortality in adults aged 36–49 years and with cardiovascular mortality in both age groups, with stronger associations observed in older participants. Significant associations with cardiovascular mortality were also seen among females, Non-Hispanic White and Black participants, current smokers, and individuals with hypertension, hyperlipidemia, cardiovascular disease, or impaired glucose tolerance. Notably, significant associations with cardiovascular mortality were found only among HSV-1–positive participants, whereas no similar associations were observed among HSV-2–positive participants (Table 3).

**Table 3:** Stratified analysis of Biological age acceleration and mortality.

	All-cause mortality	Cardiovascular mortality
Biological age acceleration (each 5-year increase)	HR (95% CI) P for interaction	HR (95% CI) P for interaction
Age (years) group		
20 - 35	1.10 (1.00, 1.20) 0.0586	1.36 (1.13, 1.63) 0.0010
36 - 49	1.09 (1.04, 1.14) 0.0002	1.20 (1.10, 1.30) <0.0001
Sex		
Male	1.04 (0.98, 1.09) 0.1684	1.12 (1.02, 1.23) 0.0230
Female	0.99 (0.92, 1.07) 0.8499	1.23 (1.06, 1.43) 0.0065
Ethnicity		
Non-Hispanic White	1.03 (0.96, 1.10) 0.3951	1.18 (1.01, 1.39) 0.0391
Non-Hispanic Black	1.02 (0.95, 1.09) 0.6632	1.18 (1.04, 1.33) 0.0079
Mexican American	0.93 (0.84, 1.03) 0.1770	0.91 (0.76, 1.10) 0.3376
Other Hispanic	0.91 (0.70, 1.18) 0.4819	0.99 (0.57, 1.70) 0.9610
Other Race	0.93 (0.74, 1.16) 0.5103	0.95 (0.64, 1.41) 0.7929
Marital status		
Married/Living with Partner	1.09 (1.03, 1.15) 0.0028	1.30 (1.17, 1.45) <0.0001
Widowed/Divorced/Separated	0.93 (0.84, 1.02) 0.1314	0.98 (0.81, 1.17) 0.7983
Never married	0.85 (0.78, 0.94) 0.0010	0.90 (0.74, 1.09) 0.2648
PIR		
Poor	0.94 (0.88, 1.02) 0.1365	1.21 (1.05, 1.40) 0.0100
Nearly poor	0.99 (0.91, 1.07) 0.7481	1.12 (0.96, 1.31) 0.1551
Middle income	1.00 (0.91, 1.09) 0.9937	1.02 (0.85, 1.24) 0.8249
High income	1.05 (0.92, 1.19) 0.5120	0.86 (0.62, 1.19) 0.3661
Education level		
Below high school	1.01 (0.89, 1.13) 0.9338	1.27 (0.99, 1.63) 0.0602
High school	0.99 (0.93, 1.05) 0.7285	1.05 (0.94, 1.18) 0.3612
Above high school	1.00 (0.93, 1.07) 0.9252	1.17 (1.01, 1.36) 0.0354
Smoking status		
Never	1.03 (0.96, 1.11) 0.3921	1.11 (0.96, 1.28) 0.1533
Former	1.03 (0.93, 1.15) 0.5568	0.86 (0.66, 1.10) 0.2251
Now	0.99 (0.93, 1.05) 0.7092	1.21 (1.08, 1.35) 0.0009
Alcohol use		
Never	0.93 (0.79, 1.09) 0.3572	1.20 (0.90, 1.59) 0.2159
Former	1.07 (0.97, 1.17) 0.1606	1.18 (0.98, 1.40) 0.0738
Mild	1.03 (0.93, 1.14) 0.5270	1.13 (0.92, 1.38) 0.2354
Moderate	1.00 (0.88, 1.13) 0.9612	1.18 (0.93, 1.51) 0.1784
Heavy	0.98 (0.91, 1.05) 0.6112	1.04 (0.89, 1.20) 0.6442
Total physical activity (MET/week)		
<600	0.94 (0.87, 1.02) 0.1289	1.03 (0.85, 1.23) 0.7779
≥600	1.06 (1.00, 1.13) 0.0588	1.11 (0.98, 1.27) 0.1089
Missing	0.97 (0.89, 1.05) 0.4212	1.18 (1.03, 1.36) 0.0159
Hypertension		
No	0.90 (0.85, 0.96) 0.0007	1.00 (0.87, 1.15) 0.9708
Yes	1.04 (0.98, 1.10) 0.1962	1.09 (0.98, 1.20) 0.1083
Diabetes Mellitus		
No	0.96 (0.92, 1.01) 0.1659	1.04 (0.93, 1.16) 0.5382
Yes	1.04 (0.96, 1.13) 0.3388	1.10 (0.96, 1.25) 0.1653
IFG	0.83 (0.68, 1.00) 0.0484	0.91 (0.64, 1.31) 0.6204
IGT	1.14 (0.82, 1.60) 0.4415	2.22 (1.17, 4.22) 0.0148
Hyperlipidemia		
No	0.96 (0.88, 1.04) 0.2964	1.02 (0.85, 1.23) 0.8155
Yes	1.01 (0.96, 1.06) 0.7361	1.14 (1.03, 1.25) 0.0081
Cardiovascular Diseases		
No	0.99 (0.94, 1.03) 0.5483	1.14 (1.04, 1.25) 0.0055
Yes	1.08 (0.98, 1.20) 0.1141	1.04 (0.88, 1.23) 0.6409
BMI (kg/m2) group		
Normal (15.02–25.39)	0.93 (0.86, 1.01) 0.0741	1.02 (0.84, 1.23) 0.8601
Overweight (25.4–30.62)	1.07 (0.99, 1.15) 0.1117	1.20 (1.04, 1.39) 0.0139
Obese (30.63–130.21)	0.99 (0.92, 1.06) 0.7593	1.08 (0.94, 1.24) 0.2631
HSV-1		
No	1.04 (0.93, 1.17) 0.4958	1.01 (0.79, 1.28) 0.9618
Yes	0.99 (0.95, 1.04) 0.7884	1.14 (1.04, 1.24) 0.0051
HSV-2		
No	1.03 (0.98, 1.09) 0.2187	1.15 (1.03, 1.28) 0.0099
Yes	0.94 (0.88, 1.01) 0.0945	1.07 (0.94, 1.22) 0.3032

Hazard ratios (HRs) and 95% confidence intervals (CIs) were estimated using multivariable Cox proportional hazards models within each subgroup. P values for interaction were calculated to test the statistical significance of effect modification across strata. All models were adjusted for age, sex, ethnicity, marital status, poverty income ratio, education level, smoking status, alcohol use, total physical activity (MET-min/week), hypertension, and diabetes mellitus. BMI body mass: index, PIR: Poverty income ratio, IFG: Impaired Fasting Glycaemia, IGT: Impaired Glucose Tolerance, HSV: Herpes simplex viruses.

In contrast, Phenotypic age acceleration showed consistent associations with both all-cause and cardiovascular mortality across most subgroups. The associations were particularly strong in participants aged 36–49 years, males, and those with cardiometabolic risk factors, including hypertension, diabetes, and impaired fasting glycemia. These patterns were robust across BMI categories, physical activity levels, and socioeconomic strata. Both HSV-1 and HSV-2–positive participants showed significant associations, with slightly stronger effects in the HSV-2 group (Table 4).

**Table 4:** Stratified analysis of phenotypic age acceleration and mortality.

	All-cause mortality	Cardiovascular mortality
Phenotypic age acceleration (each 5-year increase)	HR (95% CI) P for interaction	HR (95% CI) P for interaction
Age (years) group		
20 - 35	1.40 (1.16, 1.68) 0.0004	1.30 (0.85, 1.99) 0.2193
36 - 49	1.76 (1.61, 1.93) <0.0001	1.93 (1.63, 2.29) <0.0001
Sex		
Male	1.95 (1.74, 2.18) <0.0001	2.11 (1.72, 2.58) <0.0001
Female	1.51 (1.34, 1.71) <0.0001	1.62 (1.22, 2.15) 0.0008
Ethnicity		
Non-Hispanic White	1.84 (1.62, 2.10) <0.0001	2.26 (1.67, 3.05) <0.0001
Non-Hispanic Black	1.71 (1.49, 1.97) <0.0001	2.02 (1.57, 2.58) <0.0001
Mexican American	1.41 (1.17, 1.71) 0.0004	1.31 (0.90, 1.91) 0.1646
Other Hispanic	1.24 (0.71, 2.17) 0.4393	0.69 (0.20, 2.44) 0.5636
Other Race	1.04 (0.61, 1.76) 0.8820	0.80 (0.29, 2.16) 0.6575
Marital status		
Married/Living with Partner	1.70 (1.52, 1.90) <0.0001	1.68 (1.32, 2.14) <0.0001
Widowed/Divorced/Separated	1.53 (1.28, 1.83) <0.0001	1.85 (1.35, 2.53) 0.0001
Never married	1.60 (1.34, 1.91) <0.0001	1.84 (1.31, 2.60) 0.0005
PIR		
Poor	1.84 (1.60, 2.12) <0.0001	2.33 (1.76, 3.08) <0.0001
Nearly poor	1.51 (1.28, 1.80) <0.0001	1.55 (1.13, 2.12) 0.0061
Middle income	1.66 (1.40, 1.97) <0.0001	1.82 (1.30, 2.54) 0.0004
High income	1.51 (1.15, 1.98) 0.0028	1.88 (1.12, 3.16) 0.0176
Education level		
Below high school	1.47 (1.18, 1.84) 0.0006	1.43 (0.84, 2.43) 0.1844
High school	1.63 (1.44, 1.83) <0.0001	1.69 (1.35, 2.13) <0.0001
Above high school	1.79 (1.56, 2.05) <0.0001	2.11 (1.62, 2.77) <0.0001
Smoking status		
Never	1.42 (1.22, 1.66) <0.0001	1.50 (1.11, 2.02) 0.0088
Former	1.66 (1.33, 2.08) <0.0001	1.73 (1.14, 2.63) 0.0106
Now	1.71 (1.51, 1.92) <0.0001	1.99 (1.57, 2.52) <0.0001
Alcohol use		
Never	1.62 (1.21, 2.17) 0.0011	1.65 (0.98, 2.76) 0.0571
Former	1.76 (1.49, 2.07) <0.0001	1.98 (1.42, 2.75) <0.0001
Mild	1.49 (1.22, 1.83) 0.0001	1.51 (0.99, 2.31) 0.0558
Moderate	1.38 (1.05, 1.82) 0.0197	1.25 (0.68, 2.31) 0.4709
Heavy	1.82 (1.57, 2.11) <0.0001	2.21 (1.68, 2.91) <0.0001
Total physical activity (MET/week)		
<600	1.52 (1.31, 1.76) <0.0001	1.33 (0.94, 1.90) 0.1088
≥600	1.64 (1.42, 1.90) <0.0001	1.78 (1.34, 2.36) <0.0001
Missing	1.86 (1.62, 2.14) <0.0001	2.16 (1.70, 2.75) <0.0001
Hypertension		
No	1.44 (1.28, 1.62) <0.0001	1.44 (1.09, 1.91) 0.0099
Yes	1.75 (1.55, 1.97) <0.0001	1.78 (1.45, 2.19) <0.0001
Diabetes Mellitus		
No	1.67 (1.50, 1.86) <0.0001	1.60 (1.26, 2.03) <0.0001
Yes	1.70 (1.40, 2.06) <0.0001	1.79 (1.30, 2.47) 0.0003
IFG	2.26 (1.58, 3.24) <0.0001	3.69 (1.78, 7.67) 0.0005
IGT	1.31 (0.60, 2.86) 0.4969	1.72 (0.58, 5.08) 0.3246
Hyperlipidemia		
No	1.77 (1.50, 2.08) <0.0001	1.58 (1.11, 2.26) 0.0122
Yes	1.64 (1.49, 1.81) <0.0001	1.88 (1.56, 2.27) <0.0001
Cardiovascular Diseases		
No	1.62 (1.49, 1.77) <0.0001	1.70 (1.41, 2.06) <0.0001
Yes	1.65 (1.31, 2.08) <0.0001	1.72 (1.22, 2.42) 0.0018
BMI (kg/m2) group		
Normal (15.02–25.39)	1.72 (1.46, 2.02) <0.0001	1.56 (1.05, 2.33) 0.0289
Overweight (25.4–30.62)	1.61 (1.37, 1.89) <0.0001	1.64 (1.23, 2.19) 0.0008
Obese (30.63–130.21)	1.63 (1.41, 1.88) <0.0001	2.01 (1.54, 2.62) <0.0001
HSV-1		
No	1.58 (1.27, 1.96) <0.0001	1.87 (1.23, 2.85) 0.0034
Yes	1.68 (1.54, 1.84) <0.0001	1.80 (1.51, 2.15) <0.0001
HSV-2		
No	1.77 (1.59, 1.97) <0.0001	1.82 (1.46, 2.26) <0.0001
Yes	1.50 (1.32, 1.72) <0.0001	1.76 (1.37, 2.26) <0.0001

Hazard ratios (HRs) and 95% confidence intervals (CIs) were estimated using multivariable Cox proportional hazards models within each subgroup. P values for interaction were calculated to test the statistical significance of effect modification across strata. All models were adjusted for age, sex, ethnicity, marital status, poverty income ratio, education level, smoking status, alcohol use, total physical activity (MET-min/week), hypertension, and diabetes mellitus. BMI, body mass: index; PIR, Poverty income ratio; IFG, impaired fasting glycaemia; IGT, impaired glucose tolerance; HSV, herpes simplex viruses.

## 4. Discussion

In this cohort of HSV-positive adults in the United States, we found that biological age acceleration was associated with an increased risk of both all-cause and cardiovascular mortality. However, phenotypic age acceleration demonstrated stronger and more consistent associations with mortality outcomes compared to KDM-based biological age acceleration.

Our findings are broadly consistent with previous studies reporting associations between biological age acceleration and mortality risk in general populations. Several analyses have demonstrated that composite aging metrics, such as Phenotypic age acceleration and KDM-based biological age acceleration, are independently associated with mortality beyond chronological age [21]. PA, originally developed by Levine et al. through mortality-oriented modeling, has been shown to outperform chronological age in predicting adverse health outcomes [12]. In our analysis, Phenotypic age acceleration was significantly associated with increased risk of all-cause mortality (HR: 1.68, 95% CI: 1.47–1.92, *P* < 0.001). This effect size is comparable to that reported in the UK Biobank, where each 1-year increase in age-adjusted PA was linked to a 9% elevated risk of adverse outcomes [12]. The slightly stronger association observed in our HSV-positive population may reflect a higher degree of biological vulnerability or chronic immune activation in this subgroup.

One of the novel aspects of our study is the identification of non-linear threshold associations, particularly for Phenotypic age acceleration. Whereas many previous studies have treated biological aging as a linear covariate or have arbitrarily categorized age acceleration, we employed penalized spline and piecewise Cox regression approaches. These methods enabled us to detect that mortality risk remained relatively unchanged at lower levels of Phenotypic age acceleration, but increased substantially beyond specific inflection points. This pattern is biologically plausible and aligns with the concept of diminishing physiological reserve [22,23]. Individuals whose estimated biological age is lower than their CA may benefit from physiological resilience, whereas those with higher BA may experience heightened vulnerability [24,25]. This turning point may represent a shift from subclinical physiological variation to a state where accumulated biological damage begins to meaningfully influence mortality risk.

BA is multidimensional, and its association with health outcomes may be non-linear. Therefore, the non-linear patterns observed here should be interpreted as cohort- and metric-specific rather than as universal properties of aging. Importantly, “age acceleration” reflects the residual deviation from CA, and its biological meaning and per-unit scaling depend on how a given metric is constructed and calibrated. For example, PA is mortality-calibrated within a Gompertz framework, whereas BA is aligned to CA through biomarker regressions. Accordingly, per-unit effects and any apparent thresholds are not directly comparable across platforms and should be interpreted with caution [11,12,17].

The differing associations observed between Phenotypic age acceleration and KDM-based biological age acceleration may reflect the distinct conceptual frameworks on which they are based [12,17]. PA was constructed to capture patterns related to mortality risk by integrating multiple physiological indicators associated with systemic integrity and homeostasis, and was calibrated using a mortality-focused modeling approach. This formulation may capture biological perturbations related to subclinical inflammation and metabolic dysregulation, which have also been implicated in herpesvirus-associated immune alterations [26–29]. By contrast, the KDM approach was developed to closely align with chronological age by minimizing deviations between estimated and actual age, and incorporates a broader set of general physiological measures [30]. As a result, KDM-based estimates may be less responsive to early or transient shifts in physiological functioning, particularly in younger adults [11]. Accordingly, Phenotypic age acceleration may better reflect acute or subclinical physiological vulnerability, while KDM-based biological age acceleration captures longer-term deviations in age-related physiological integrity [31].

Although our study was not designed to establish causality, the observed associations raise important questions regarding the potential role of HSV infection in biological aging. Chronic herpesvirus infections, particularly those prone to frequent reactivation, may contribute to systemic inflammation and immune exhaustion, both of which are implicated in accelerated biological aging [32]. Prior studies have reported that elevated HSV antibody titers are associated with increased mortality risk among older adults, potentially reflecting cumulative immune burden [33]. In our HSV-positive cohort, however, we observed substantial heterogeneity in biological age acceleration, as measured by both Phenotypic age acceleration and KDM-based biological age acceleration. Notably, a considerable proportion of individuals showed negative phenotypic age acceleration values, suggesting that their BA was younger than their CA. These findings suggest that HSV infection alone is unlikely to account for the full spectrum of BA observed in this population. Future research should investigate whether viral load, reactivation frequency, or co-infections further influence biological age acceleration trajectories among HSV-infected individuals.

Our study benefits from a large, nationally representative sample and robust mortality linkage, as well as the use of two complementary aging metrics and flexible modeling techniques. We also adjusted for a comprehensive set of demographic, socioeconomic, and clinical covariates. Nevertheless, limitations must be acknowledged. First, although mortality data were longitudinally linked, BA was assessed at a single time point, limiting inference about aging trajectories. Second, our cohort was relatively young (mostly under 50), which may limit applicability to older HSV-infected adults. Third, our study lacked an HSV-negative control group, and therefore we could not assess whether HSV modifies the strength of the association between biological age acceleration and mortality. To partially address this issue, we conducted supplementary stratified analyses by HSV-1 and HSV-2 status within the seropositive cohort (Supplementary Figures 1). These exploratory analyses suggested potential differences in the associations between biological aging measures and mortality, but the reduced sample size limited statistical stability. Therefore, our conclusions cannot be generalized to HSV-negative populations, and future studies including both HSV-positive and HSV-negative individuals are needed to clarify whether herpesvirus infection modifies the trajectory of biological aging. Another limitation is that NHANES provides serological data on HSV-1 and HSV-2 infection but does not systematically capture clinical reactivation history (e.g., oral cold sores, genital lesions, or recurrence frequency). Thus, we could not distinguish whether the associations observed differ between asymptomatic latent infection and recurrent disease. Future studies incorporating detailed clinical information are warranted. Finally, residual confounding from unmeasured variables cannot be excluded. In addition, many participants had comorbidities and external factors such as alcohol use and low socioeconomic status that may influence biological age acceleration. Although we adjusted for a comprehensive set of covariates, residual confounding from these and other unmeasured variables cannot be entirely excluded, and this further limits interpretability.

In addition to these considerations, it is important to clarify our choice of aging models. We focused on KDM-based BA and PA because these measures are reproducible, validated, and derived from standardized NHANES biomarkers. KDM captures deviations from age-expected norms, whereas PA predicts mortality risk, providing complementary perspectives. Alternative models such as Allostatic Load and Homeostatic Dysregulation rely on population-specific thresholds, which may reduce generalizability in large-scale epidemiological studies.

## 5. Conclusion

In cohort of HSV-seropositive adults in the United States, biological age acceleration, particularly phenotypic age acceleration, was significantly associated with increased risks of all-cause and cardiovascular mortality.

## Supporting information

S1 Fig HSV-1 and HSV-2 infection status within the HSV-seropositive population. (DOCX)

S1 Table Supplementary data. (XLS)
