# Supplementary figures and images for: Association of biological age acceleration with all-cause and cardiovascular mortality in HSV-positive adults: A population-based longitudinal cohort study
Source: PLoS One. 2025 Oct 14;20(10):e0334621. doi: 10.1371/journal.pone.0334621 (PMC12520405; doi:10.1371/journal.pone.0334621)

Supplementary Figures 1:

**
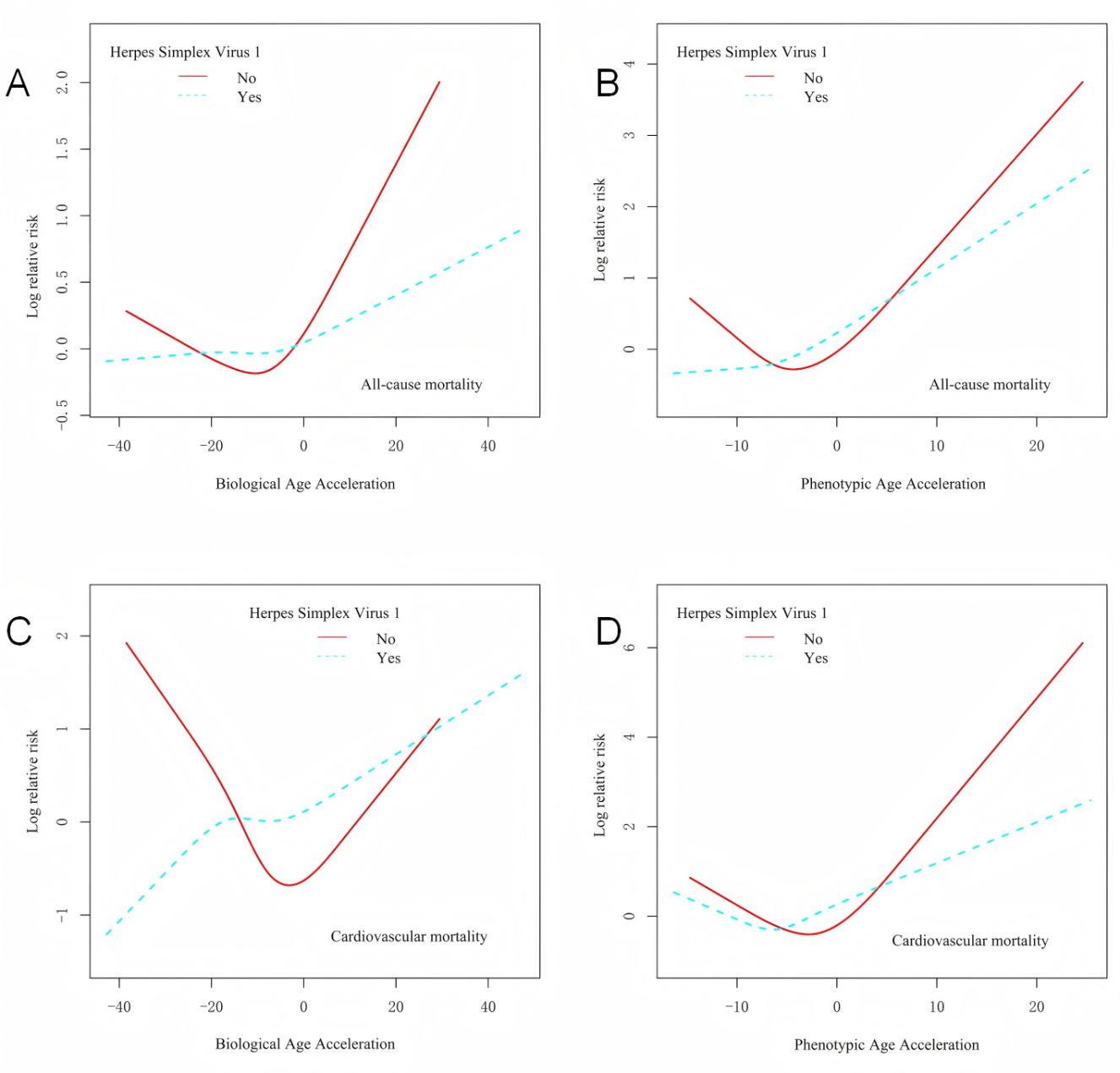
**


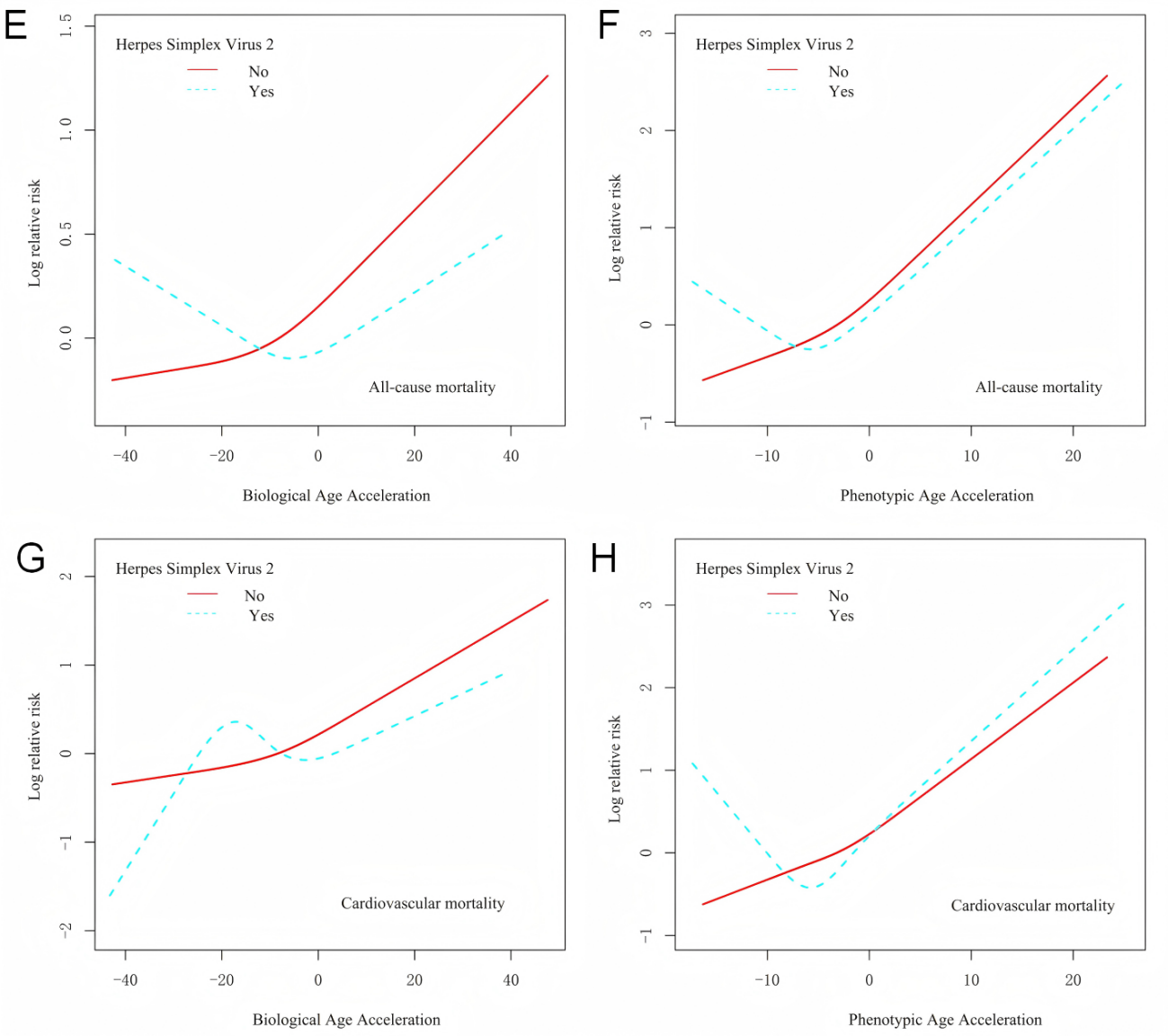

Supplement: S1 Fig — (DOCX) [file pone.0334621.s001.docx]
